# Geohelminth Infections and Nutritional Status of Preschool Aged Children in a Periurban Settlement of* Ogun State*

**DOI:** 10.1155/2016/7897351

**Published:** 2016-02-29

**Authors:** O. O. Omitola, H. O. Mogaji, A. S. Oluwole, A. A. Adeniran, O. M. Alabi, U. F. Ekpo

**Affiliations:** Department of Pure and Applied Zoology, Federal University of Agriculture, PMB 2240, Abeokuta, Nigeria

## Abstract

This study assessed the geohelminth and nutritional status of preschoolers in a periurban community of Ogun state. Fresh stool specimens were collected for laboratory analysis, processed using ether concentration method, and examined under the microscope for geohelminth ova. Demographic characteristics and daily nutrient intake of children were subjectively assessed during an interview session with parents, following anthropometric data collection. Data obtained were analysed using a statistical software for Windows. Nutritional indicators such as underweight, stunting, and wasting were computed from anthropometric data. Results showed an overall prevalence of 39.2% and 12.4% for Ascariasis and Hookworm infection, respectively, with no significant difference (*P* > 0.05) between the sexes. Prevalence of nutritional indicators was 52.6%, 35.1%, 34.0%, and 9.3% for underweight, stunting, wasting, and thinness conditions, respectively. A good proportion of the malnourished preschoolers were free of* Ascaris* infection but infected with Hookworm parasite. The adverse effect of geohelminth infection cannot still be ignored in impaired growth, reduced survival, poor development, and cognitive performance of preschoolers. Therefore promotion of adequate health education program on measures of preventing geohelminth infections is needed.

## 1. Introduction

Geohelminth infections are common in tropical and subtropical regions of the developing world especially in Sub-Saharan Africa (SSA), where poor domestic and environmental hygiene prevails [1]. Nigeria, the most populous country in SSA, is endemic for geohelminth infections due to ascariasis, trichuriasis, and hookworm with estimated cases of 55 million, 34 million, and 38 million, respectively [2–4]. Favourable edaphic and climatic conditions contribute to the development of the Geohelminth, while inadequate sanitation facilities, lack of safe drinking water source, poor nutrition, and overcrowding are factors aiding their transmission [5, 6]. Decades back, Stephenson and Holland highlighted the public health importance of these infections in alteration of normal human gastrointestinal flora and aggravation of other nutritional disturbances either by reducing food intake and/or increasing nutrient wastage via vomiting, diarrhea, or blood loss [7]. The poor nutritional status or malnutrition due to these infections has also been implicated in poor cognitive functioning of preschool aged children when enrolled in schools [8, 9]. Malnutrition affects the physical, mental, and social wellbeing and development of children, and it has been an underlying cause of over half of child deaths in many developing countries [10, 11]. Nevertheless, the pool of Geohelminth infection related studies in Nigeria has focused on investigating prevalence and intensity [12–14], with little information on the impact of geohelminth infections on the nutritional status of their host, especially among preschool and school aged children [15]. However recent findings presented the impact of these parasites on nutritional status of school aged children [15]. This study however surveyed geohelminth infection and the nutritional status of preschool aged children in a periurban settlement of Ogun state, Nigeria.

## 2. Materials and Methods

### 2.1. Study Area

This study was carried out in a purposively selected periurban community “Isale-Ijeun” located within Abeokuta South Local Government Area of Ogun state, Nigeria. The inhabitants primarily depend on public taps for water resource and dug wells as supplement. Proper hygiene and sanitation practices and waste disposal facilities are however lacking in these areas.

### 2.2. Participant and Inclusion Criteria

Community members of the selected area were sensitized about the research through the assistance of Primary Health Care (PHC) centre staffs and the Community Development Association (CDA) leaders. A total sampling of households within the community was done and consenting parents with children belonging to age category 0–6 years were recruited into the study in accordance with Ekpo et al. [16]. Parents of children who could provide adequate stool specimen at the point of visit were asked to converge at the PHC centre where detailed anthropometric measurements of their children and interviews were made.

### 2.3. Research Ethics

The research protocol was introduced to the community leader through the already consented Primary Health Care (PHC) Coordinator. The research idea was then later translated to the community members (parents and caregivers) through the community development association (CDA). Informed consent forms were verbally translated to the parents and caregiver in their local language; only parents/caregivers who consented by signing the consent form were recruited into the study.

### 2.4. Collection of Stool Samples

A labeled sterile plastic universal sample bottle was given to each consenting parent/caregiver. Freshly voided stool specimens of the preschool children were collected at the point of visit from the parent/caregiver. Samples were transferred to the laboratory within two hours of collection.

### 2.5. Questionnaire Administration

Parents/caregivers whose children could provide adequate stool specimen were carefully interviewed using a close-structured questionnaire at the PHC centre. Demographic information of each child, nutrient intake, household sanitary, and personal hygiene conditions were documented using the questionnaire.

### 2.6. Anthropometric Measurement

Anthropometric indices such as weight, height, and age were recorded for each child providing stool sample. Weight was measured in kilogrammes (kg) using the domestic HAMSON® bathroom weighing scale. Weights of most infants (0-1 years) were however collected from most recent neonatal data on their Neonatal Medical Record Cards. Weights of others were measured by weighing them together with their parents while carrying them and deducting the parent's weight from the weight measured. Height (and length for infants) was measured using the common builder's measuring tape and age was collected by enquiry from parents/caregivers.

### 2.7. Laboratory Analysis of Stool Specimen

One gram of the stool sample collected from each preschooler was prepared using SAF (Sodium-Acetate-Acetic Acid-Formalin) ether concentration technique to increase the sensitivity of STH ova detection. Samples were emulsified in 10 mL of SAF solution and transported in ice packs to the Parasitology laboratory, Department of Pure and Applied Zoology, for analysis within two hours of collection. In the laboratory, sample bottles were vigorously agitated to efficiently suspend the stool in the solution. Stool suspension was further strained through a 13 mm sieve into a centrifuge tube, and the filtrate was recentrifuged at 2000 rpm for 5 minutes. The resulting supernatant was discarded; then 7 mL of normal saline and 3 mL of petroleum ether were added to the sediment. The resulting mixture was shaken vigorously and centrifuged for 5 minutes at 2000 rpm. The first three layers of the suspension observed after centrifuging were discarded leaving the last layer of sediment. Sediment was pipetted onto a clean, oil free glass slide and examined for the ova of gastrointestinal helminths under ×10 objective lens [17].

### 2.8. Anthropometric/Nutritional Analysis

Nutritional analyses were carried out using Height-for-Age (HAZ), Weight-for-Age (WAZ), and BMI-for-height (BMZ) obtained from weight, height, and age data according to WHO Child Growth Standard using the WHO Child Growth Standards SPSS Syntax File for SPSS (2007) for the data for children of age of 0–5 years and the WHO Reference 2007 SPSS macropackage (2008) for the data for children of age 6. Children whose HAZ, WAZ, and BMZ were above −2 SD scores were considered normal and those below −2 SD and −3 SD scores were considered malnourished and severely malnourished, respectively [18, 19].

### 2.9. Statistical Data Analysis

All statistical analyses were performed using IBM SPSS 20.0 version, Armonk, NY, IBM Corp. Data obtained were first subjected to descriptive statistics including frequencies and cross-tabulations, followed by Pearson chi-square analysis to test for variables that were significantly associated with infection and malnutrition among the surveyed population. Factors that showed some significant relationship (*P* values < 0.05) with helminth infection and malnutrition were selected as potential risk factors into our models for predictive analysis using logistic regression. Potential risk factors were entered into the model as covariates using bidirectional stepwise entry method. Reference category was formulated for categorical variables before analysis and observations with missing values for any variable were excluded from the analysis. Predictive index in the model is represented as Exp(*B*).

## 3. Result

### 3.1. Demography of the Preschoolers

A total of 190 preschool aged children were recruited into the study, but only 97 could provide adequate stool samples for microscopic examination. Corresponding demographic data analysed from the 97 (100%) surveyed preschool children revealed that a subtotal of 53 (54.6%) were males and 44 (45.4%) were females. Also, majority of these preschool children 81 (83.5%) were with the age range of 0–60 months while 16 (16.5%) of them were above 60 months old (Table 1).

### 3.2. Prevalence of Geohelminths

Table 2 shows the prevalence of geohelminths infection among the surveyed preschool aged children. An overall prevalence of 39.2% and 12.4% was recorded for ascariasis and hookworm infections while 43.3% prevalence was recorded for coinfection of both geohelminths.

### 3.3. Prevalence of Geohelminths by Sex and Age

The distribution of these geohelminths by sex across the surveyed preschool children was not significantly different (*P* > 0.05), though 20 males (20.6%) were more infected with* Ascaris lumbricoides* infections than females 18 (18.6%). But females 7 (7.2%) were more infected with hookworm than males 5 (5.2%). However, geohelminth infections were higher and significantly different (*P* < 0.05) for ascariasis and the coinfections among preschool aged children within 0–60-month age category and children within 60–72-month category (Table 3).

### 3.4. Nutrient Intake of the Preschoolers

The nutrient intakes of the preschool aged children were summarized in Table 4. Majority of the children (94) (96.9%) consume either fish or meat daily as their source of protein. However, other daily diet includes carbohydrate, milk, and egg with 74 (76.3%), 19 (19.6%), and 2 (2.1%) respondents, respectively.

### 3.5. Nutritional Status of the Preschoolers

Table 5 shows the nutritional status of the preschool aged children. Overall prevalence of 35.1%, 52.6%, 34.0%, and 9.3% was recorded for stunting, underweight, wasting, and thinness conditions, respectively, among the preschoolers. However, 13.4%, 33.0%, 18.6%, and 1.0% of the preschoolers had severe condition of stunting, underweight, wasting, and thinness, respectively.

### 3.6. Nutritional Status of the Preschoolers by Sex and Age

Across all the nutritional indicators, males were more adversely affected than females with stunting (19.6% versus 15.1%), underweight (30.9% versus 21.6%), and thinness (5.2% versus 4.1%) for males and females, respectively. However there were no significant differences (*P* > 0.05) in malnutrition indices recorded by sex category except for thinness (*P* < 0.050). By age category, children younger than 60 months old were more malnourished compared to those older than 60 months with stunting prevalence of (26.8% versus 8.2%), underweight (42.3% versus 10.3%), wasting (34.0% versus 0), and thinness (0 versus 9.3%) for children within age category of 0–60 months and 60–72 months, respectively (Table 6).

### 3.7. Nutritional Status and Geohelminth Infections among Preschoolers

Majority of the malnourished preschoolers were not infected with* Ascaris lumbricoides*, with malnutrition prevalence of 18.6% versus 16.5% for stunting, 27.8% versus 24.7% for underweight, and 23.7% versus 10.3% for wasting conditions between the infected and noninfected preschoolers, respectively, though there was no significant difference (*P* > 0.05) across both categories. However, preschoolers infected with* Ascaris lumbricoides* were more malnourished for thinness condition compared to noninfected preschoolers with 6.2% and 3.1%, respectively. For hookworm infections, infected preschoolers were more malnourished than noninfected preschoolers for stunting and underweight conditions with 26.8% versus 8.2% and 42.3% versus 10.3%, respectively, though there was no significant difference (*P* > 0.05) (Table 7).

### 3.8. Water Source and Sanitary Conditions of Preschoolers

All the preschool aged children obtain their daily water from both public tap and hand-dug well water sources; however majority of them (70) (72.2%) use open pit latrine and 19 (19.6%) of them defecates on open fields. Among those that use toilet facilities, 76 (78.4%) of them share with another member of their household or community (Table 8).

### 3.9. Hygiene, Attitude, and Practice of Preschoolers

76 (78.3%) of the preschool children wash their hands before eating, though with water alone as reported by 72 (74.2%) of them. However of the 97 respondents only 18 (18.6%) wash their hands after defecation. Playing on soil, picking objects on the soils, and lack of sandals when playing on soil were also reported by 84 (86.6%), 90 (92.8), and 78 (80.4) preschool aged children, respectively (Table 9).

### 3.10. Prevalence of Geohelminth Infections and Associated Risk Factors

Table 10 presents the association between geohelminth infection and the risk factors documented among the preschoolers. Infection generally with* Ascaris lumbricoides* (32) (33%) and Hookworms (8) (8.2%) was common among preschoolers using open pits as toilet type. Preschoolers that shared toilet facilities with household members were also more infected with* Ascaris lumbricoides* (32) (33%) and hookworms (8) (8.2%) compared to those that do not. In addition, preschoolers that wash their hand with soap were less infected compared to those that wash with water or do not wash at all either before eating or defecating. Playing on soils, not wearing sandals, and picking food from the ground were also found to be more common among preschoolers infected with* Ascaris lumbricoides* and hookworm (Table 10).

### 3.11. Predictive Factors for Helminth Infection among the Surveyed Children

Parameters with probability values less than 0.05 (age group, washing of hands before eating, playing on soil, and wearing of sandals) were selected into our logistic regression models accordingly to predict relationships with helminth infections (Table 11).

From our results, the maximum likelihood models predicted that the selected parameters will improve the fit of the models. Omnibus test of coefficients also showed that the logistic models were fit. However, none of the risk factors selected was a significant predictor in our model for* Ascaris* infection. On the other hand,* wearing of sandals* was a significant predictor for* Ascaris* and Hookworm coinfection and *R* ^2^ analysis attributed 15.9% variation in helminth infection to these two factors.

Children older than 60 months were thrice at risk of* Ascaris* and hookworm coinfection than those below 60 months. Children wearing sandals were less likely to report* Ascaris* and hookworm coinfection with an odds ratio of 0.07.

### 3.12. Predictive Analysis for Malnutrition among the Surveyed Children

Parameters with probability values less than 0.05 (sex and* Ascaris* infection) were selected into our logistic regression models accordingly to predict relationships with malnutrition indicators (Table 12).

Omnibus test showed that our logistic regression model for malnutrition was not fit. While chi-square test identified sex and* Ascaris* infection as potential risk factors for some malnutrition indices (weight-for-height and BMI-for-age), they were not significantly predictive coefficients in our logistic regression model.

## 4. Discussion

The prevalence of 39.2% and 12.4% reported for ascariasis and hookworm infection in this study reflects the vulnerability of preschool aged children to geohelminth infections and justifies the need to include this age group in deworming programmes. Although this prevalence is lower compared to that of [20] where prevalence of 65.2% and 48.7% was reported for ascariasis and hookworm infection, respectively, the differences in prevalence might be attributed to timing, seasonal differences when conducting the survey, sample size, study group, geographic factors of study area, poor sanitation, and personal and environmental hygiene in study area and among children surveyed [6].

*Ascaris lumbricoides* had the highest prevalence in this study followed by hookworm and these findings corroborate with that of [20, 21]. The development and transmission of this geohelminth indicate that soil, food, and water might be contaminated with infective stages. Though the study area had better water conditions, however, the prevalent sanitary conditions of the locality are such that favours the development and transmission of the parasites. Poor hygienic practices of not washing hands with soaps after defaecation and attitudes such as picking food from ground, playing on soil, and more importantly not wearing sandals might have predisposed them to the risk of contaminating their hands with infective stages of* Ascaris lumbricoides* ova and also active penetration of infective hookworm larvae on soil as the case may be.

Potential risk factors such as washing of hand, wearing of sandals, and playing on soil identified in our study were not significant predictors for* Ascaris* infection. Our expectation may have been affected by insufficient data on these parameters. However, the age of the preschoolers was a significant predictive factor for infection with* Ascaris* and hookworm infections in this study. The sampled children became more prone to helminth infections as they grew older. In such environment with poor sanitary conditions, this may be attributed to less dependence on parents in the older children as identified by [22, 23] who reported similar trends and attributed it to weaning from breast milk and better mobility. Hence the important role of parents in aiding preschoolers towards practicing personal and domestic hygiene such as wearing of sandals which is a significant protective factor for* Ascaris* and hookworm coinfection in this study should not be undermined.

The overall prevalence of 52.6%, 35.1%, 34.0%, and 9.3% recorded in this study for underweight, stunting, wasting, and thinness conditions as indicators of nutritional status among the surveyed preschool aged children is of great public health concern. This prevalence is higher compared to that reported by [9] in a study conducted in Uganda where 5.3% of the children examined were underweight, 22.5% were stunted, and 18.5% were wasted. Also studies conducted in Ethiopia, China, and India reported lower stunting prevalence of 26.5%, 25.6%, and 37% for the children surveyed, compared to the stunting prevalence in this study [24–26]. However, the prevalence of 52.6% reported for underweight conditions in this study is in similitude with those reported by [26, 27] where 51.7% and 60.9% of the Indian school children surveyed were underweight. Reason for incoherent malnutrition indices prevalence between this study and other studies may be attributed to differences in geohelminth prevalence of the study area. In addition, dietary inadequacies among the children surveyed might be implicated in the prevalence of malnutrition indicators reported in this study [15]. Though majority of the preschoolers reportedly consume fish or meat as a daily source of protein, however the subjective method used in assessing nutrient-intake of the preschool aged children from their parents or guardians needs to be validated as respondents might overreport or underreport situations which might be as a result of length of recall or disclosure issues as usually observed in recall surveys [28]. As important as daily intake of protein source is to children growth and development, sustaining this feeding habit in extreme rural or poor settings where parents/caregivers are either unemployed, peasant farmers, or low income earners is a challenge. There is therefore an important need to educate parent and caregivers on preventive measures such as avoiding contact with soil, avoiding open defecation, wearing of sandals, and good hand hygiene such as cutting of nails and washing of hands with water and soap at the critical times.

A good proportion of the malnourished preschoolers reported in this study were free of* Ascaris* infection but infected with Hookworm parasite. Although the causes of malnutrition are multifactorial, our finding deviates from the expected. Also, predictive analysis by logistic regression showed that our malnutrition model is not fit, and selected potential risk factors in this study (sex and* Ascaris* infection) were not protective. This could be explained in that thresholds might exist for geohelminth infections before impacting on nutritional status of infected individuals when other factors are controlled. Nevertheless, the adverse effect of geohelminth infection cannot still be ignored in impaired growth, reduced survival, poor development, and cognitive performance of preschool children even before enrolling into schools [10, 11].

The analysis of risk factors in this study was based on the presence or absence (prevalence) and not burden (intensity) of infection, although this is in similitude with the investigation of [22]; the lack of information on the burden of infection with helminths among our study participants is a limitation for further regression analysis. Future investigations with similar focus should therefore consider the estimation of helminths burden among preschoolers.

Also, only 15.9% variation in our helminth prevalence data could be accounted for by risk factors documented in this study. It therefore appears that other important covariates are not accounted for in this study. Hence, further studies are needed to document important predisposing risk factors and identify their predictive or protective strengths for helminth infection and malnutrition indices among preschoolers.

## Figures and Tables

**Table 1:** Demographic characteristic of the surveyed preschool aged children.

Variables	Number of respondents (%)
Sex	
Male	53 (54.6)
Female	44 (45.4)
Total	97 (100)
Age range (months)	
0–60	81 (83.5)
60–72	16 (16.5)
Total	97 (100)

**Table 2:** Prevalence of geohelminths infection among the surveyed preschool aged children.

	Number examined	Number infected (%)
*Ascaris lumbricoides* only	97	38 (39.2)
Hookworm only	97	12 (12.4)
*Ascaris lumbricoides* + hookworm	97	42 (43.3)

**Table 3:** Prevalence of geohelminth infection by sex and age group across the preschool aged children examined.

	*Ascaris lumbricoides* infection	Hookworm infection	*Ascaris lumbricoides* + hookworm infection
	Number infected (%)	Number infected (%)	Number infected (%)
Sex			
Male	20 (20.6)	5 (5.2)	21 (21.6)
Female	18 (18.6)	7 (7.2)	21 (21.6)
Total	38 (39.2)	12 (12.4)	42 (43.3)
*P* value	0.750	0.335	0.423
Age range (in months)			
0–60	27 (27.8)	10 (10.3)	31 (32.0)
60 and above	11 (11.3)	2 (2.1)	11 (11.3)
Total	38 (39.2)	12 (12.4)	42 (43.3)
*P* value	0.000	0.436	0.001

**Table 4:** Summary of nutrient intake of the preschool aged children surveyed.

	Number of respondents	Percentages
Eat meat/fish daily		
Yes	94	96.9
No	1	1.0
Not applicable	2	2.1
Daily frequency of eating meat/fish		
Eat three times daily	2	2.1
Eat more than three times daily	93	95.9
Not applicable	2	2.1
Other daily diet		
Milk	19	19.6
Egg	2	2.1
Carbohydrate	74	76.3
Not applicable	2	2.1

**Table 5:** Nutritional indices (*Z*-score) of the surveyed preschool aged children.

	Nutritional Indices			
	Height-for-age (stunting)	Weight-for-age (underweight)	Weight-for-height (wasting)^*∗*^	BMI-for-age (thinness)^*∗∗*^
Number examined	97	97	81	16
Number below −2 SD (acute malnutrition)	34	51	33	9
% below –2 SD	35.1	52.6	34.0	9.3
Number below −3 SD (severe malnutrition)	13	32	18	1
% below –3 SD	13.4	33.0	18.6	1.0
Overall number of malnourished preschoolers	34	51	33	9
Overall % of malnourished preschoolers	35.1	52.6	34.0	9.3

*∗*: recorded for children less than 5 years of age (0–60 months old)

*∗∗*: recorded for children above 5 years of age (60–72 months old).

**Table 6:** Nutritional indices (*Z*-score) of the surveyed preschool aged children by sex and age category.

	Nutritional Indices			
	Height-for-age (stunting)	Weight-for-age (underweight)	Weight-for-height (wasting)^*∗*^	BMI-for-age (thinness)^*∗∗*^
Number examined	97	97	81	16
Sex				
Male	19 (19.6%)	30 (30.9%)	18 (18.6%)	5 (5.2%)
Female	15 (15.1%)	21 (21.6%)	15 (15.5%)	4 (4.1%)
Total	34 (35.1%)	51 (52.6%)	33 (34.0%)	9 (9.3%)
*P* value	0.857	0.383	0.169	0.042
Age				
0–60 months	26 (26.8%)	41 (42.3%)	33 (34.0%)	0 (0%)
60–72 months	8 (8.2%)	10 (10.3%)	0 (0%)	9 (9.3%)
Total	34 (35.1%)	51 (52.6%)	33 (34.0%)	9 (9.3%)
*P* value	0.170	0.384	0.000	0.000

*∗*: recorded for children less than 5 years of age (0–60 months old)

*∗∗*: recorded for children above 5 years of age (60–72 months old).

**Table 7:** Nutritional indices (*Z*-score) and geohelminth infections of the surveyed preschool aged children.

	Nutritional Indices			
	Height-for-age (stunting)	Weight-for-age (underweight)	Weight-for-height (wasting)^*∗*^	BMI-for-age (thinness)^*∗∗*^
Number examined	97	97	81	16
*Ascaris lumbricoides* infection				
Number infected	16 (16.5%)	24 (24.7%)	10 (10.3%)	6 (6.2%)
Not infected	18 (18.6%)	27 (27.8%)	23 (23.7%)	3 (3.1%)
Total	34 (35.1%)	51 (52.6%)	33 (34.0%)	9 (9.3%)
*P* value	0.243	0.094	0.027	0.029
Hookworm infection				
Number infected	26 (26.8%)	41 (42.3%)	6 (6.2%)	2 (2.1%)
Not infected	8 (8.2%)	10 (10.3%)	27 (27.8%)	7 (7.2%)
Total	34 (35.1%)	51 (52.6%)	33 (34.0%)	9 (9.3%)
*P* value	0.894	0.296	0.417	0.408
*Ascaris* and Hookworm infections				
Number infected	17 (17.5%)	26 (26.8%)	12 (12.4%)	6 (6.2%)
Not infected	17 (17.5%)	25 (25.8%)	21 (21.6%)	3 (3.1%)
Total	34 (35.1%)	51 (52.6%)	33 (34.0%)	9 (9.3%)
*P* value	0.328	0.108	0.077	0.078

*∗*: recorded for children less than 5 years of age (0–60 months old)

*∗∗*: recorded for children above 5 years of age (60–72 months old).

**Table 8:** Water and sanitary conditions of the surveyed preschool aged children.

	Number of respondents	Percentages
Water source in use		
Hand-dug well	—	—
Public tap	—	—
Both	97	100
Toilet type in use		
Open pit	70	72.2
Pit with slab	5	5.2
Water closet	3	3.1
Bush	19	19.6
Shared usage of toilet		
Yes	76	78.4
No	2	2.1
Not applicable	19	19.6

**Table 9:** Hygiene attitudes and practices of the surveyed preschool aged children.

	Number of respondents	Percentages
Washing of hands after defecation		
Yes	18	18.6
No	79	81.4
If Yes, with what		
Water	14	14.4
Water and soap	4	4.1
Not applicable	79	81.4
Washing of hands before eating		
Yes	76	78.3
No	21	21.6
If Yes, with what		
Water	72	74.2
Water and soap	4	4.1
Not applicable	21	21.6
Wearing of sandal when playing		
Yes	17	17.5
No	78	80.4
Not applicable	2	2.1
Picking objects from the ground		
Yes	90	92.8
No	5	5.2
Not applicable	2	2.1
Playing on soil with friends		
Yes	84	86.6
No	11	11.3
Not applicable	2	2.1

**Table 10:** Prevalence of geohelminth infections and associated risk factors.

	*Ascaris lumbricoides* infection	Hookworm	Ascaris + hookworm infection
Toilet type in use			
Open pit	32 (33%)	8 (8.2%)	35 (36.1%)
Pit with slab	0 (0)	0 (0)	0 (0)
Water closet	1 (1%)	0 (0)	1 (1%)
Bush	5 (5.2%)	4 (4.1%)	6 (6.2%)
Total	38 (39.2)	12 (12.4%)	42 (43.3%)
*P* value	0.120	0.474	0.099
Shared usage of toilet			
Yes	32 (33%)	8 (8.2%)	35 (36.1%)
No	1 (1%)	0 (0)	1 (1%)
Not applicable	5 (5.2%)	4 (4.1%)	6 (6.2%)
Total	38 (39.2%)	12 (12.4%)	42 (43.3%)
*P* value	0.429	0.398	0.513
Washing of hand before eating or defecation			
Yes, with water	10 (10.3%)	2 (2.1%)	10 (10.3%)
Yes, with water and soap	1 (1%)	0 (0)	1 (1%)
Not applicable	27 (27.8%)	10 (10.3%)	31 (32.0%)
Total	38 (39.2%)	12 (12.4%)	42 (43.3%)
*P* value	0.026	0.734	0.061
Wearing of sandal when playing			
Yes	1 (1%)	0 (0)	1 (1%)
No	36 (37.1%)	12 (12.4%)	40 (41.2%)
Not applicable	1 (1%)	0 (0)	1 (1%)
Total	38 (39.2)	12 (12.4)	42 (43.3)
*P* value	0.008	0.189	0.003
Picking objects from the ground			
Yes	37 (38.1%)	12 (12.4%)	41 (42.3%)
No	0 (0)	0 (0)	0 (0)
Not applicable	1 (1.0%)	0 (0)	1 (1%)
Total	38 (39.2)	12 (12.4)	42 (43.3)
*P* value	0.177	0.587	0.133
Playing on soil with friends			
Yes	37 (38.1%)	11 (11.3%)	40 (41.2%)
No	0 (0)	1 (1%)	1 (1%)
Not applicable	1 (1%)	0 (0)	1 (1%)
Total	38 (39.2)	12 (12.4)	42 (43.3)
*P* value	0.018	0.806	0.052

**Table 11:** Logistic regression models for helminth infection and* Ascaris* infection.

	Helminth infection			*Ascaris* infection		
	Exp(*B*)	S.E	*P* value	Exp(*B*)	S.E	*P* value
Intercept model	0.76	0.21	0.19	0.64	0.21	^*∗*^0.03
Logistic model^*∗∗*^						
Age group						
0–60 months	Reference	—	—	Reference	—	—
≥60 months	3.35	0.51	^*∗*^0.02	4.10	0.54	^*∗*^0.01
Playing on soil with friends						
No				Reference	—	—
Yes				9.83E7	1.19E4	0.99
Wearing of sandals when playing						
No	Reference	—	—	Reference	—	—
Yes	0.09	1.07	^*∗*^0.02	0.32	1.13	0.31
Washing of hand before eating						
No				Reference	—	—
Water				4.66	1.11	0.16
Water + soap				0.00	2.70E4	0.99

^*∗∗*^Cox and Snell *R* ^2^ = 0.159 (*Ascaris* and hookworm) and 0.243 (*Ascaris*)

^*∗*^ *P* value < 0.05 shows significant predictive factor.

**Table 12:** Logistic regression models for malnutrition indices.

	*B*	Weight-for-height (WHZ)			*B*	BMI-for-age (BMZ)		
		Exp(*B*)	S.E	*P*-value		Exp(*B*)	S.E	*P*-value
Intercept model	0.375	0.688	0.23	0.098	0.251	1.29	0.50	0.62
Logistic model^*∗∗*^								
Sex								
Female					Reference	—	—	—
Male					21.680	2.60E7	2.00E4	0.99
*Ascaris *infection								
Negative	Reference	—	—	—	Reference	—	—	—
Positive	0.23	1.26	0.48	0.63	−0.47	0.63	1.40	0.74

^*∗∗*^Cox and Snell *R* ^2^ = 0.000 (WTH) and 0.302 (BMZ)

^*∗*^ *P* value < 0.05 shows significant predictive factor.

## Limitation of the Study

The present study has a low sample size because only one community was surveyed. In addition, no attempt was made to quantitatively measure the parasitic burden of geohelminth infections or determine the prevalence of other infections (i.e., protozoan infections) among the preschoolers. Therefore, the result from this study can only be used to generalize with caution.
